# 7p22.3 microdeletion: a case study of a patient with congenital heart defect, neurodevelopmental delay and epilepsy

**DOI:** 10.1186/s13023-024-03321-8

**Published:** 2024-08-16

**Authors:** Liliya Skvortsova, Anastassiya Perfilyeva, Kira Bespalova, Yelena Kuzovleva, Nailya Kabysheva, Ozada Khamdiyeva

**Affiliations:** 1 Laboratory of Molecular Genetics, Institute of Genetics and Physiology, Almaty, 050060 Kazakhstan; 2 Department of Molecular Biology and Genetics, Al-Farabi Kazakh National University, Almaty, 050040 Kazakhstan

**Keywords:** 7p22.3 deletion, Ventricular septal defect, Autism, Epilepsy

## Abstract

**Background:**

Chromosome 7 has regions enriched with low copy repeats (LCRs), which increase the likelihood of chromosomal microdeletion disorders. Documented microdeletion disorders on chromosome 7 include both well-known Williams syndrome and more rare cases. It is noteworthy that most cases of various microdeletions are characterized by phenotypic signs of neuropsychological developmental disorders, which, however, have a different genetic origin. The localization of the microdeletions, the genes included in the region, as well as the structural features of the sequences of these genes have a cumulative influence on the phenotypic characteristics of the individuals for each specific case and the severity of the manifestations of disorders. The consideration of these features and their detailed analysis is important for a correct and comprehensive assessment of the disease.

**Results:**

The article describes a clinical case of 7p22.3 microdeletion in a patient with congenital heart defect and neurological abnormalities - epilepsy, combined with moderate mental and motor developmental delay.

**Conclusions:**

Through detailed genetic analyses, we are improving the clinical description of the rare 7p22.3 microdeletion and thus creating a basis for future genetic counseling and research into targeted therapies.

## Background

A 7p22.3 microdeletion is a rare genetic condition where a small piece of chromosome 7 is missing. This deletion can result in various health issues depending on the specific genes that are involved. Microdeletion 7p22.3 is a rare genetic anomaly characterized by the absence of a segment on chromosome 7 and leads to various health complications depending on the genes affected. Some reported features associated with this microdeletion include developmental delays, intellectual disability, growth problems, and certain facial characteristics. The severity and combination of symptoms may differ, but neurodevelopment delays and heart defects are the most consistent features. Recently, Marbach F and colleagues reported direct evidence between the *PRKAR1B* gene, located on 7p22.3, and certain types of neurodevelopmental delays [1]. The relationships between the 7p22.3 microdeletion and heart defects are less clear than neurodevelopmental delays, and more research is needed to establish a specific correlation. The comparative analysis of published cases with the deletion in the 7p22.3 locus, identified a minimal region causative for heart malformations [2, 3]. There are two potential candidate genes *SNX8* and *EIF3B* responsible for the heart malformations development in this region. Involvement of these genes in cardiac embryonic development is not fully understood and requires additional experimental and clinical cases.

Here, we describe a one-year-old girl with a 2.4 Mb deletion at the 7p22.3 locus involving 40 genes that shows learning and language delay, epilepsy, autism, hypotonia, dyspraxia and ventricular septal defect.

## Materials and methods

### Clinical presentation and family history

**Fig. 1:**
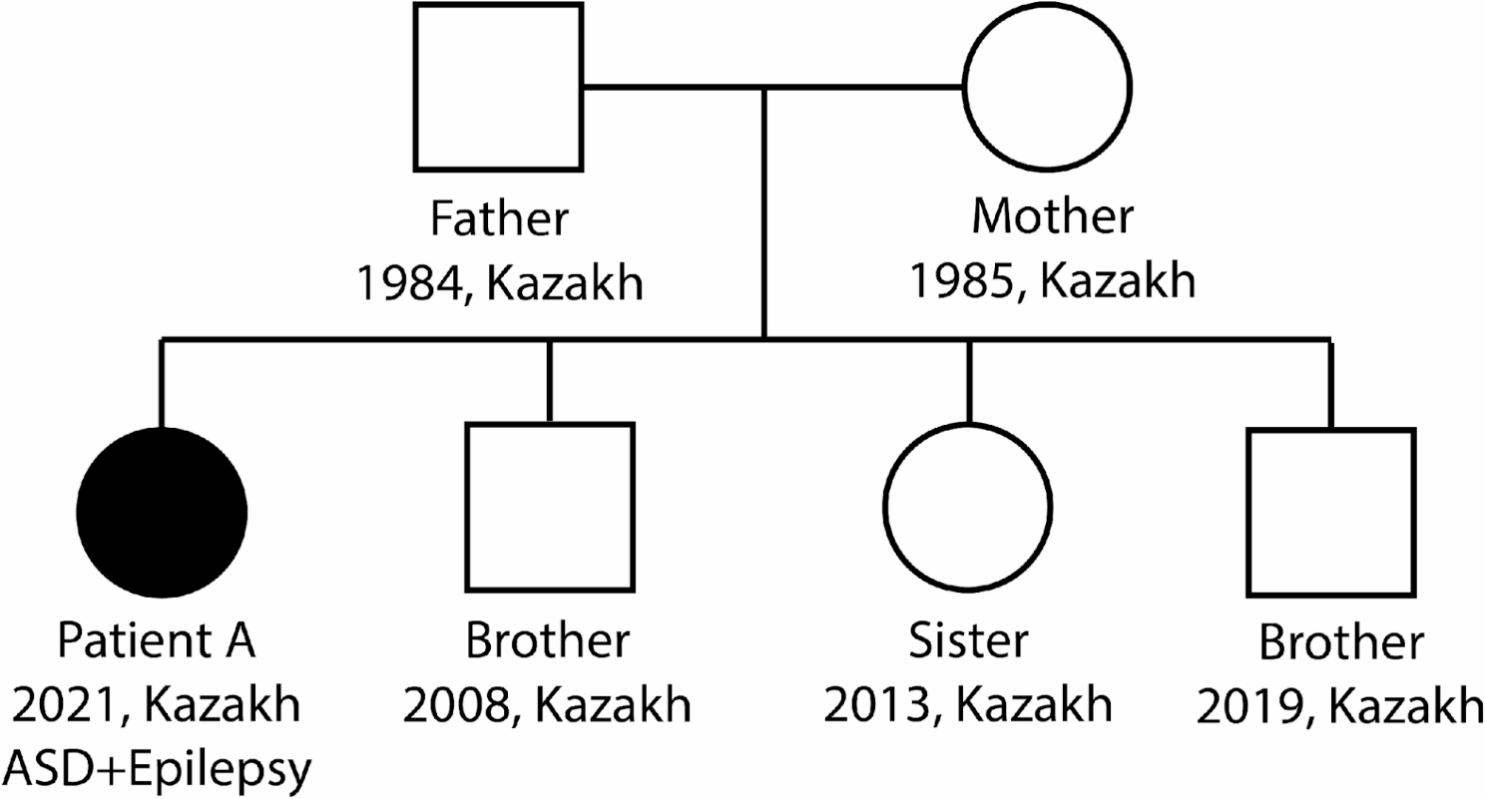
Pedigree of the family included in the case study

The patient A is a 1 year 6 months old girl of Kazakh nationality, born to non consanguineous parents. The child from the 4th pregnancy of a mother with a history of endocrine disorder and apparently healthy father (Fig. 1). Body weight is 10 kg and height is 79 cm (Z-score = 0.1; percentile = 54.8), the head circumference is 44 cm (Z-score =−1.6; percentile = 5.3); her face and palpebral fissures are symmetrical, the tongue lies in the midline. Muscle tone is severely reduced symmetrically, relative to the sagittal axis (D = S). Muscle strength is reduced, support on the feet is weakened - support on the toes when standing. In addition, there is a developmental coordination disorder - dyspraxia. History of epileptic tonic seizures; remission for 6 months while taking the levetiracetam therapy. She does not chew solid food or drink from a cup (drink is squirted into her mouth with a syringe).

The contact is unproductive due to ignoring her name or requests/instructions; She doesn’t make eye contact and her reaction of joint attention is negative. She is fenced off and extremely attached to her smartphone, in which she independently finds and plays fragments of cartoons. She is not interested in toys and prefers to manipulate non-play objects. Her emotions are inexpressive, monotonous and it is extremely difficult to attract her attention. She reacts positively only to a tactile stimulation, pronounces sounds and babbling elements without the purpose of the communication. Her behavior is autonomous. There are no self-service and neatness skills.

### Birth, growth and neurologic history

The girl was born at 38 weeks of gestation via normal, spontaneous vaginal delivery. The birth weight was 2983 g and the length was 50 cm. She cried out immediately, the skin and limbs of her body were bluish in color and the Apgar score was 4–7 points. A diagnosis of congenital heart defect, ventricular septal defect (VSD) was verified. The child was breastfed from birth and showed difficulty in latching and sucking with subsequent vomiting and regurgitation. Due to the increased pulmonary hypertension, VSD surgery was done at the age of 2 months to correct a hole between the left and right ventricles of the heart. The thymus was removed during the VSD surgery. At 7 months of age, single tonic seizures appeared during teething and a low-grade fever. Subsequently, tonic seizures were noted at the body temperature of 38 °C (frequent acute respiratory viral infections due to removal of the thymus) in the form of prolonged tension in the limbs and increased sweating. The tonic febrile seizures did not disappear after the cessation of the underlying illness and epilepsy with tonic seizures were diagnosed by a neurologist.

The child began to hold her head at 4 months, to sit at 12 months, to crawl at 13 months and to walk with support at 15 months. Onomatopoeia was noted in the child from 6 months and babbling was noted from 10 months. She pronounces individual syllables, knows her family and sleeps peacefully.

The patient is the fourth child of four and her three siblings are unaffected (Fig. 1). The family lives in an environmentally unfavorable region of Kazakhstan, with high technogenic pollutants from industrial plants producing salt, coal and other minerals.

### Chromosome microarray analysis (CMA)

The СMA was performed at the Centre for Molecular Medicine (Almaty, Kazakhstan). Array CGH was performed using 4 × 180 K microarrays from Oxford Gene Technology (CytoSure ISCA, v3, Oxford, UK). All genomic coordinates were based on the reference genome (NCBI37/hg19). Data analysis was performed using CytoSure Interpret Software (Oxford Gene Technology, Oxford, UK) and the circular binary segmentation algorithm. The calling thresholds were deviation of a circular binary segmentation (CBS) segment from zero log ratio of + 0.30 for duplications and−0.5 for deletions. The results were then classified using CytoSure Interpret Software (Oxford Gene Technology, Oxford, UK). Quality control metrics were monitored using CytoSure Interpret Software (Oxford Gene Technology).

### DNA extraction

DNA was extracted with Promega Wizard™ Genomic DNA Purification Kits according to the manufacturer’s protocol and quantified with Qubit.

### Whole exome sequencing (WES), sequencing read alignment and variant calling

DNA samples were sequenced by Celemics, Inc (Seoul, South Korea) on the Illumina NovaSeq Sequencing Platform. Sequencing reads were aligned to the GRCh37 reference genome using BWA mem v0.7.17 [4]. Aligned reads were sorted into the format BAM using SAMtools v.1.15.1 [5]. Following preprocessing steps included marking duplicates and Base Quality Score Recalibration (BQSR) using both GATK v4.3.0.0 tools MarkDuplicates and BaseRecalibrator/ApplyBQSR, respectively [6]. GATK v4.3.0.0 HaplotypeCaller was used to generate raw variants (SNPs and InDels) [7]. Quality Score Recalibration (VQSR) was applied to distinguish true genetic variants from artifacts using GATK v4.3.0.0 tools VariantRecalibrator/ApplyVQSR. The raw SNPs and Indels were filtered using GATK v4.3.0.0 tool VariantFiltration with parameters “QD < 2.0, FS > 60.0, MQ < 40.0, MQRankSum <−12.5, ReadPosRankSum <−8.0, SOR > 4.0” and “QD < 2.0, FS > 200.0, ReadPosRankSum <−20.0, SOR > 10.0”, respectively. Using posterior genotype probabilities, possible de novo mutations were marked with the GATK v4.3.0.0 tool VariantAnnotator. The SNPSift/SNPEff and GATK v4.3.0.0 tool Funcotator was used for the annotation of variants [8].

The resulting sets of variants were filtered by excluding a splice region variant, a synonymous variant, a coding transcript intron variant, a non-coding transcript intron variant, an upstream gene variant, a downstream gene variant, an intergenic variant and a regulatory region variant. The filtered variants were further prioritized according to minor allele frequency (MAF ≤ 1%), pathogenicity, inheritance pattern and phenotype data. MAFs were determined using data from the 1000 Genomes Project https://www.internationalgenome.org/ and the Genome Aggregation Database https://gnomad.broadinstitute.org/. The pathogenic potential and phenotype concordance of the identified variants were assessed using the ClinVar database (https://gnomad.broadinstitute.org/). In-silico predictions of the pathogenicity of the variants were performed using Sorting Intolerant From Tolerant (SIFT, https://sift.bii.a-star.edu.sg/), Polymorphism Phenotyping v2 (PolyPhen−2, https://genetics.bwh.harvard.edu/pph2/) and MutationTaster (https://www.mutationtaster.org/). The Exomiser programme and VarElect tool was additionally used to prioritize the variants [9, 10].

## Results

### Molecular cytogenetic examination

**Fig. 2:**
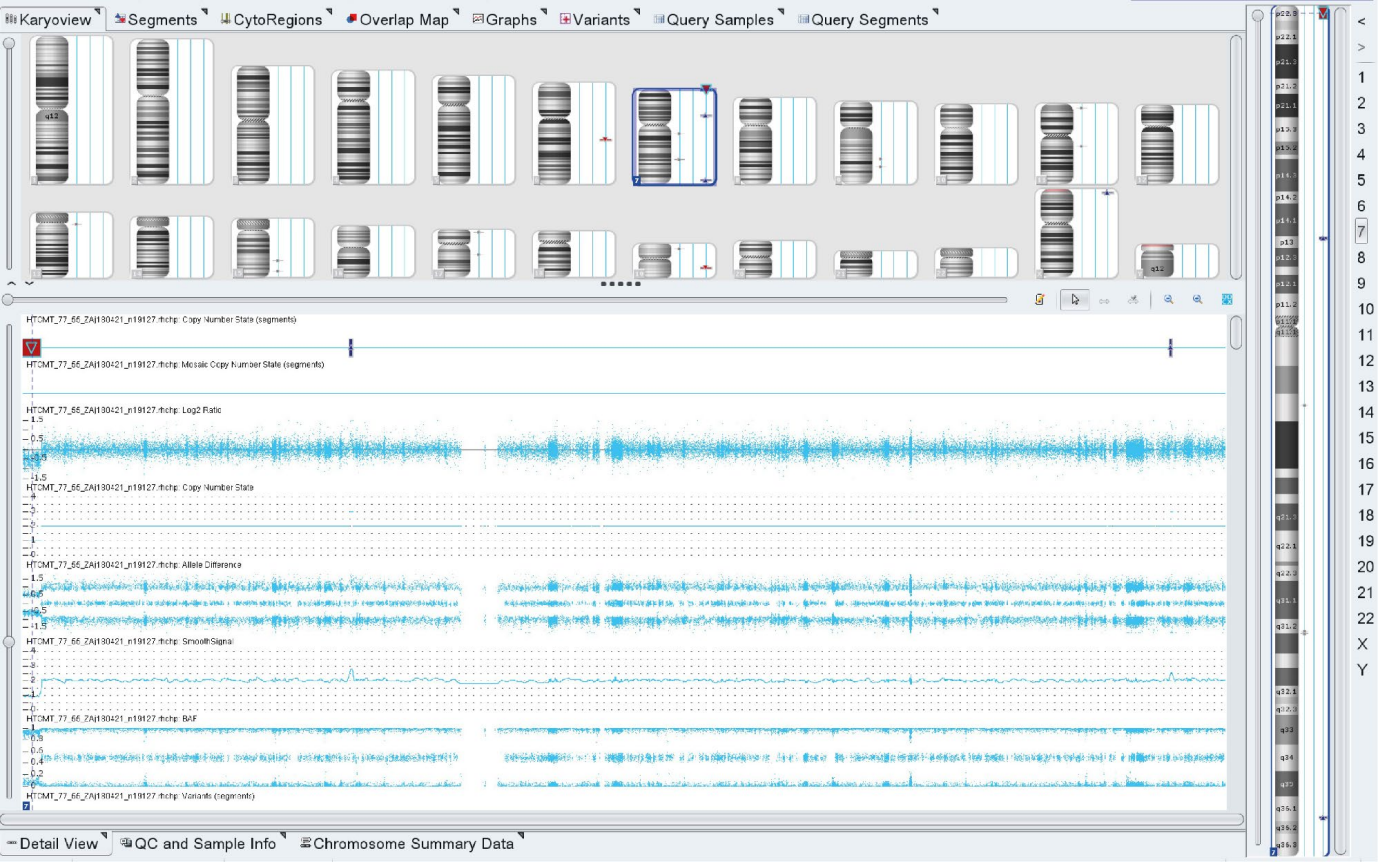
Results of the CMA. Deletion of 2.4 Mb in the 7p22.3 region ranging from 22,118 to 2,434,088 bp (hg19)

Given the severe early postnatal medical history and the presence of a number of congenital phenotypic characteristics, it was assumed that the patient had structural chromosomal rearrangements/chromosomal abnormalities. Chromosome microarray analysis (CMA) was performed to confirm the genetic alterations at the chromosomal level. Based on the CMA results, the patient’s molecular karyotype arr[GRCh38] 7p22.3(XX)×1 was determined, confirming the presence of a microdeletion of 2,411,970 bp on the short arm of the chromosome 7 (Fig. 2).

**Fig. 3:**
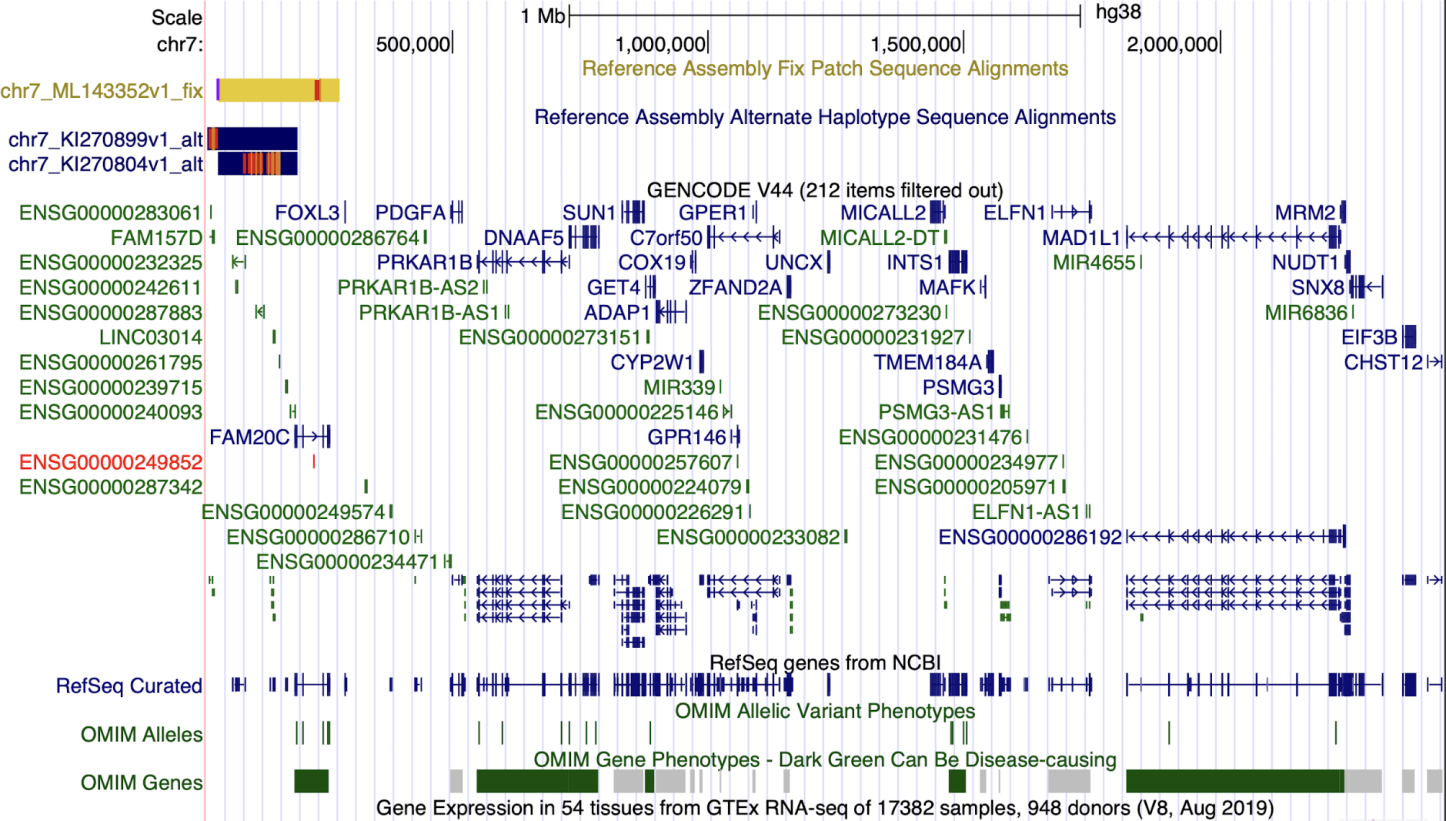
Deleted genes in 7p22.3 region detailed by Genome Browser (UCSC)

The locus of imbalance includes 40 genes, which are shown in Fig. 3. In the Decipher and ClinVar databases, microdeletions of this region are described as likely pathogenic and/or variants of unknown clinical significance (VUS) in patients with minor anomalies, congenital defects and developmental delays. The detected microdeletion is not indexed in the DGV database of normal genomic variants. Microdeletions in this region are identified in the Orphanet database as a distal monosomy 7p (ORPHA:96126). Therefore, the identified microdeletion can be considered as VUS.

### Diagnostic testing

At the age of 16 months, the patient A was referred for a diagnosis of orphan diseases by tandem mass spectrometry. According to the results, no abnormalities were found in the levels of amino acids, carnitines, lysophospholipids, nucleosides, ketones, or enzymes.

An additional test of the spectrum of organic acids in the urine using gas chromatography (60 indicators) to identify congenital metabolic disorders showed no deviations from normal values.

### Whole exome sequencing

For a thorough analysis of the patient’s genetic profile, whole exome sequencing (WES) was performed on patient A, as well as on their father, mother and healthy sister. A total of over 159 million reads were generated using an Illumina NovaSeq sequencer for the family of patient A, covering approximately 48,265,177,638 base pairs. Table 1 contains summarized statistics for all reads. Of these, 591,582 single nucleotide variations (SNVs) and 98,423 insertions/deletions (InDels) were identified, from which 475,093 SNVs and 90,042 InDels met the criteria for successful filtering. A total of 344 high confidence *de novo* mutations were detected, which were absent in the healthy sister but present in the patient A. A total of 4,901 variants corresponded to an autosomal recessive inheritance pattern. The variants were annotated and classified according to the standards of the American College of Medical Genetics and Genomics. All variants were assessed for evidence of pathogenicity in the ClinVar database. No variants associated with the development of epilepsy in patient A were identified.

**Table 1:** Summarized statistics for WES reads

ID	Read type	No. of reads	No. of total bases (bp)	Mean depth over target region
Patient A	paired-end	34,450,164	10,403,949,528	109.01
Mother	paired-end	40,519,576	12,236,911,952	144.17
Father	paired-end	41,433,242	12,512,839,084	136.18
Sister	paired-end	43,415,487	13,111,477,074	141.26

## Discussion

Complex disease phenotypes affecting different organ systems are the result of an individual’s complex genetic landscape. Analysis of the literature data shows that there is a certain variability of phenotypes even in Mendelian diseases. Symptoms may vary in the presence/absence and severity from patient to patient. These individual phenotypes are the most noticeable in microdeletion syndromes when multiple genes are affected. One of these syndromes is Williams syndrome, which is caused by a heterozygous deletion 7q11.23 [11, 12]. There are specific symptoms and features with a high frequency of occurrence (core symptoms, > 90% of cases) and less specific features with a frequency of < 90% (Morris 2023). The last ones are highly variable from patient to patient. This emphasizes that microdeletion syndromes are not a uniform disease but a group of etiologically heterogeneous conditions whose pathophysiology reflect an individual genetic profile. The functional activity of affected proteins and their involvement in cellular processes as well as the presence of additional structural changes in single-copy genes modulate a pathological phenotype.

In the present case, the patient A had most of the symptoms typical for Williams syndrome and was referred for a genetic counseling (to be confirmed by genetic testing). CMA revealed the absence of the specific microdeletion in the long arm of chromosome 7 (7q11.23) but the presence of another microdeletion in the short arm of chromosome 7 (7p22.3). The identified deletion is less common than the Williams syndrome deletion and some cases have been published. Deletion 7p22.3 phenotype is associated with various clinical symptoms including neurodevelopmental delay, congenital skeletal and heart abnormalities, hypotonia, craniofacial dysmorphism and other less common features [3]. To date, epilepsy has not been reported for this 7p22.3 deletion but it was observed in a patient with a combination of 7p22.3-p22.1 deletion and 8q24.23-q24.3 duplication. In this case, the occurrence of epilepsy in a patient was apparently related to the *KCNQ3* gene located within the 8q24.23-q24.3 duplication [13]. It is well known that genes encoding ion channels in the brain are dosage-sensitive genes and have dominant traits [14, 15]. In contrast, epilepsy was not observed in two siblings with large 7p22.3-p22.2 and 15q11.1-q11.2 deletions, emphasising the absence of epilepsy-related genes in the 7p22.3 deletion region [16] In addition to epilepsy, the patient A had other symptoms not previously mentioned in the 7p22.3 deletion, such as microcephaly, feeding problems and autistic traits. Further detailed analysis of the 7p22.3 microdeletion region did not reveal any genes directly associated with these additional abnormalities (Table 2).

The patient has three main types of abnormalities: neurological development, epilepsy and cardiac malformation. We examined the role of each gene within the 7p22.3 deletion to describe the complex phenotype of our case.

**Table 2:** Summary of the affected genes and their associated diseases

Gene	Chr	Mutation type	Trait type	Gene OMIM	Associated diseases	Disease OMIM
*FAM20C*	7	HI	Recessive	OMIM 611,061	Raine Syndrome	OMIM 259,775
*FOXL3*	7	HI				
*PDGFA*	7	HI		OMIM 173,430		
*PRKAR1B*	7	HI	Dominant	OMIM 176,911	Marbach-Schaaf Neurodevelopmental Syndrome	OMIM 619,680
*DNAAF5*	7	HI	Recessive	OMIM 614,864	Ciliary Dyskinesia, Primary, 18	OMIM 614,874
*SUN1*	7	HI		OMIM 607,723		
*GET4*	7	HI	Recessive	OMIM 612,056	Congenital Disorder of Glycosylation, Type IIy	OMIM 614,874
*ADAP1*	7	HI		OMIM 608,114		
*COX19*	7	HI		OMIM 610,429		
*C7orf50*	7	HI				
*CYP2W1*	7	HI		OMIM 615,967		
*GPR146*	7	HI				
*GPER1*	7	HI		OMIM 601,805		
*ZFAND2A*	7	HI		OMIM 610,699		
*UNCX*	7	HI				
*MICALL2*	7	HI				
*INTS1*	7	HI	Recessive	OMIM 611,345	Neurodevelopmental Disorder with Cataracts, Poor Growth, and Dysmorphic Facies	OMIM 618,571
*MAFK*	7	HI		OMIM 600,197		
*TMEM184A*	7	HI				
*PSMG3*	7	HI		OMIM 617,528		
*ELFN1*	7	HI		OMIM 614,964		
*MAD1L1*	7	HI	Recessive	OMIM 602,686	Mosaic Variegated Aneuploidy Syndrome 7 with Inflammation and Tumor Predisposition	OMIM 620,189
*MRM2*	7	HI	Recessive	OMIM 606,906	Mitochondrial Dna Depletion Syndrome 17	OMIM 618,567
*NUDT1*	7	HI		OMIM 600,312		
*SNX8*	7	HI		OMIM 614,905		
*EIF3B*	7	HI	Recessive	OMIM 603,917		

HI – haploinsufficiency

Neurological abnormalities such as developmental delay, hypotonia and apraxia/dyspraxia found in our patient may be described by the recently identified Marbach-Schaaf Neurodevelopmental Syndrome (MASNS) associated with the *PRKAR1B* gene [1]. According to the case study by Marbach F et al., affected patients also exhibited behavioural abnormalities such as autistic and sensory-seeking behavioral. The diagnosis of autism spectrum disorder (ASD) and sensory processing disorder (sensation seeking) made by expert-physicians in our patient is consistent with these characteristics. *PRKAR1B* mutations found by Marbach F et al. are heterozygous and highlights the importance of the presence of two wildtype *PRKAR1B* gene copies for normal brain development and phenotype. The haploinsufficiency of the *PRKAR1B* gene due to the 7p22.3 deletion and the absence of pathogenic mutations in other genes within this deletion is the (primary) causative factor for the intellectual disability phenotype observed in our patient. The question of whether the deficiency of the *PRKAR1B* gene is directly involved in the development of an epilepsy remains open, as there is little evidence for this association. One patient described by Marbach F et al., had seizures, apparently familiar that the patient’s mother had a history of seizure disorder. Other important data are not available to make any precise conclusions. In other cases with a haploinsufficiency of the *PRKAR1B* gene due to the 7p22.3 region deletion, no incidence of seizures were observed.

A developing brain is a sensitive organ that can be affected by various factors. Childhood febrile seizures and delayed neurological development are risk factors for the incidence of epilepsy [17]. The mechanisms underlying this pathology are poorly understood but experimental data indicated the importance of inflammatory processes and their regulation in the brain [18, 19]. An up-regulation of inflammatory response pathways with subsequent activation of glial cells and astrocytes, as well as the production of pro-inflammatory cytokines and chemokines is observed. Additionally, neuroinflammation and the activation of the immune response caused by other neurological pathologies may be triggers for the development of epilepsy [20–22]. By shifting the balance of the local membrane microenvironment, neuroinflammation changes the activity of ion channels that are important for both normal neuronal activity (synaptic transmission, excitation-inhibition balance) and the development of seizures (excitatory-inhibitory imbalance) [23, 24]. The genetic landscape of the affected ion channels and closely related proteins also contributes to the epileptic activity of neurons.

The coincidence of two processes associated with neuroinflammation in our patient - febrile seizures and congenital neurodevelopmental abnormalities - appear to be the main factors in the development of epilepsy. Notably, our WES analysis did not reveal any significant pathogenic mutations in epilepsy-related genes. It is likely that other more common nucleotide changes in these genes may be important in the aforementioned pathologies and predispose neurons to an excitatory-inhibitory imbalance. This question is complex and requires further research.

The literature data suggest that the 7p22.3 locus is an important region for heart development, and case studies have reported congenital heart defects in patients with deletions of different sizes of 7p22. However, the specific genes responsible for the abnormal heart development are still under investigation and debate. The proposed candidate genes are *SNX8* and *EIF3B* but their clinical and experimental data are controversial. The SNX8 protein belongs to a large family of proteins involved in intracellular endosome trafficking, sorting of various proteins and cellular homeostasis through the Golgi network [25, 26]. The importance of these proteins in pathologies is not fully understood but given their ubiquitous cytoplasmic expression and low tissue specificity they may have variable expressivity and/or reduced penetrance in different organs. This controversy has been noted in congenital heart defects in cases with different sizes of 7p22.3 deletions [27]. A comparative analysis of published 7p22.3 deletion cases by Mastromoro G et al. [2] and Tessier A et al. [27] shows that *SNX8* haploinsufficiency may be necessary but not a sufficient cause of heart malformations. Another candidate gene is the *EIF3B* gene, which encodes the subunit B of the eukaryotic translation initiation factor 3 (eIF3). EIF3 is the largest and most complex translation initiation factor for proteins’ biosynthesis in cells and consists of 13 subunits (eIF3a–eIF3m) [28]. Various incoming signals and controlled downstream target mRNAs for the translation initiation make this complex an essential regulator for the development and functioning of an organism. Given the important role of eIF3, mechanisms underlying various pathologies caused by insufficiency of the eIF3 complex have been the focus of attention for many years. In particular, different subunits have been associated with different processes: the subunit e (eIF4e) was found to be more important in embryonic development [29]; the subunit a (eIF3a) in the development of brain, lungs, fat, skin, spleen, and thymus [30]; the subunit b (eIF3b) in the development of heart and nervous system [31, 32]. The available experimental data on model organisms show that the complexity of the eIF3 interactions gradually increases through evolution.

Zebrafish experiments have shown that the lack of functional activity of the eif3ba protein (eIF3b, the human ortholog) most affects cranial neural crest development, leading to dramatic changes in the cranial neural crest cells (cNCCs) migration and derivative heart tissue development [33]. Although these changes are compatible with embryonic development in zebrafish, they are lethal to post-embryonic development at day 5. For the most complex vertebrates like mice, the complete absence of the protein is lethal to the embryo at the blastocyst stage but its reduced activity was compatible with the embryonic development and not with the post embryonic development (6.5 days).

The role of cNCCs in heart formation is well established in mammalian development and abnormalities in cNCCs may affect a spectrum of congenital heart outflow tract defects (tetralogy of Fallot, ventricular septal defect, overriding aorta, double outlet of the right ventricle, persistent truncus arteriosus, and others [34, 35]. The question of how relevant downstream signaling pathways function when one copy of the eIF3b gene is lost remains open. Experimental evidence on mice suggests that heterozygosity of eif3b +/- has no visual phenotypic abnormalities because the mice were successfully crossed and the offspring were obtained.

Thus, the currently available studies do not allow to identify specific genes directly responsible for the development of heart pathologies in the presence of the microdeletion in the 7p22.3 region. Additional molecular and genetic studies are required to clarify this relationship. Moreover, in our WES analysis, we focused on variants that directly alter the amino acid sequence of proteins and excluded variants in the splicing region, synonymous variants, intronic variants, upstream and downstream gene variants, intergenic variants, and variants in the regulatory region because it is extremely difficult to predict their functional effects. Predicting the effects of splice region variants is complex due to the complicated nature of splicing mechanisms and the involvement of multiple regulatory elements, which can lead to unreliable or false positive results [36]. The functional effects of synonymous variants are also difficult to predict as they do not lead to corresponding protein sequence changes [37]. The databases used to annotate these variants may not provide complete information, which increases the risk of misinterpretation [37]. In addition, there is a problem that there are no truly negative variants, i.e. those that have been shown to have no effect on the protein function or no association with the disease [38]. In addition, WES does not capture intronic and other non-exonic regions with the same depth, resulting in incomplete data for variants in splice regions and other non-coding variants. This limitation reduces the reliability and completeness of the data for these regions. This is particularly important as many high-ranking splice variants associated with neurodevelopmental disorders such as autism have been detected intronically [39]. These findings suggest that whole genome sequencing, combined with highly effective tools for predicting the functional impact of variants that do not directly alter the amino acid sequence, would overcome the limitations of WES in detecting intronic and non-coding regions and could provide a more comprehensive understanding of the patient’s disease profile.

## Conclusions

We reported a microdeletion of 2.4 Mb in the 7p22.3 region in an individual with neurodevelopmental disorder, epilepsy and congenital heart defect. Our report confirms the role of the 7p22.3 region in neurodevelopmental delay, socio-emotional behavior and suggests further studies of potential candidate genes within the deletion in the pathogenesis of congenital heart defects and epilepsy.

## Data Availability

The raw data of the WES of the patient A (F2P), the father (F2F), the mother (F2M) and the healthy sister (F2S) are publicly available in SRA-NCBI (https://www.ncbi.nlm.nih.gov/sra/PRJNA1089791), SRA accession SRP411987.

## References

[1] Marbach F, Stoyanov G, Erger F, Stratakis CA, Settas N, London E et al. Variants in PRKAR1B cause a neurodevelopmental disorder with autism spectrum disorder, apraxia, and insensitivity to pain. Genet Med [Internet]. 2021 [cited 2024 Mar 19];23:1465–73. https://pubmed.ncbi.nlm.nih.gov/33833410/10.1038/s41436-021-01152-7 PMC8354857 33833410

[2] Mastromoro G, Capalbo A, Guido CA, Torres B, Fabbretti M, Traversa A et al. Small 7p22.3 microdeletion: Case report of Snx8 haploinsufficiency and neurological findings. Eur J Med Genet [Internet]. 2020 [cited 2024 Mar 19];63. https://pubmed.ncbi.nlm.nih.gov/31568860/10.1016/j.ejmg.2019.103772 31568860

[3] Richards EG, Zaveri HP, Wolf VL, Kang SHL, Scott DA. Delineation of a less than 200 kb minimal deleted region for cardiac malformations on chromosome 7p22. Am J Med Genet A [Internet]. 2011 [cited 2024 Mar 19];155:1729–34. https://onlinelibrary.wiley.com/doi/full/10.1002/ajmg.a.34041 10.1002/ajmg.a.34041 21671376

[4] Li H, Durbin R. Fast and accurate short read alignment with Burrows-Wheeler transform. Bioinformatics [Internet]. 2009 [cited 2024 Mar 19];25:1754–60. https://pubmed.ncbi.nlm.nih.gov/19451168/10.1093/bioinformatics/btp324 PMC2705234 19451168

[5] Li H, Handsaker B, Wysoker A, Fennell T, Ruan J, Homer N et al. The Sequence Alignment/Map format and SAMtools. Bioinformatics [Internet]. 2009 [cited 2024 Mar 19];25:2078. https://pubmed.ncbi.nlm.nih.gov/19505943/10.1093/bioinformatics/btp352 PMC2723002 19505943

[6] McKenna A, Hanna M, Banks E, Sivachenko A, Cibulskis K, Kernytsky A et al. The Genome Analysis Toolkit: a MapReduce framework for analyzing next-generation DNA sequencing data. Genome Res [Internet]. 2010 [cited 2024 Mar 19];20:1297–303. https://pubmed.ncbi.nlm.nih.gov/20644199/10.1101/gr.107524.110 PMC2928508 20644199

[7] Depristo MA, Banks E, Poplin R, Garimella KV, Maguire JR, Hartl C et al. A framework for variation discovery and genotyping using next-generation DNA sequencing data. Nature Genetics. 2011 43:5 [Internet]. 2011 [cited 2024 Mar 19];43:491–8. https://www.nature.com/articles/ng.806 10.1038/ng.806 PMC3083463 21478889

[8] Cingolani P, Platts A, Wang LL, Coon M, Nguyen T, Wang L et al. A program for annotating and predicting the effects of single nucleotide polymorphisms, SnpEff: SNPs in the genome of Drosophila melanogaster strain w1118; iso–2; iso–3. Fly (Austin) [Internet]. 2012 [cited 2024 Mar 19];6:80. https://pubmed.ncbi.nlm.nih.gov/22728672/10.4161/fly.19695 PMC3679285 22728672

[9] Stelzer G, Plaschkes I, Oz-Levi D, Alkelai A, Olender T, Zimmerman S et al. VarElect: the phenotype-based variation prioritizer of the GeneCards Suite. BMC Genomics [Internet]. 2016 [cited 2024 Mar 19];17 Suppl 2. https://pubmed.ncbi.nlm.nih.gov/27357693/10.1186/s12864-016-2722-2 PMC4928145 27357693

[10] Bone WP, Washington NL, Buske OJ, Adams DR, Davis J, Draper D et al. Computational evaluation of exome sequence data using human and model organism phenotypes improves diagnostic efficiency. Genetics in Medicine 2016 18:6 [Internet]. 2015 [cited 2024 Mar 19];18:608–17. https://www.nature.com/articles/gim2015137 10.1038/gim.2015.137 PMC4916229 26562225

[11] Baumer A, Dutly F, Balmer D, Riegel M, Tükel T, Krajewska-Walasek M et al. High level of unequal meiotic crossovers at the origin of the 22q11. 2 and 7q11.23 deletions. Hum Mol Genet [Internet]. 1998 [cited 2024 Mar 19];7:887–94. https://pubmed.ncbi.nlm.nih.gov/9536094/10.1093/hmg/7.5.887 9536094

[12] Morris CA. Williams Syndrome. GeneReviews^®^ [Internet]. 2023 [cited 2024 Mar 19]; https://www.ncbi.nlm.nih.gov/books/NBK1249/

[13] Touhami R, Foddha H, Alix E, Jalloul A, Mougou-Zerelli S, Saad A et al. Case report: 7p22.3 deletion and 8q24.3 duplication in a patient with epilepsy and psychomotor delay—Does both possibly act to modulate a candidate gene region for the patient’s phenotype? Front Genet [Internet]. 2022 [cited 2024 Mar 19];13. https://pubmed.ncbi.nlm.nih.gov/36778913/10.3389/fgene.2022.1061539 PMC9909830 36778913

[14] Roll P, Szepetowski P. Epilepsy and ionic channels. Epileptic Disorders [Internet]. 2002 [cited 2024 Mar 19];4:165–72. https://www.jle.com/en/revues/epd/e-docs/epilepsy_and_ionic_channels_110011/article.phtml?tab=texte 12446218

[15] Imbrici P, Liantonio A, Camerino GM, De Bellis M, Camerino C, Mele A et al. Therapeutic Approaches to Genetic Ion Channelopathies and Perspectives in Drug Discovery. Front Pharmacol [Internet]. 2016 [cited 2024 Mar 19];7. https://pubmed.ncbi.nlm.nih.gov/27242528/10.3389/fphar.2016.00121 PMC4861771 27242528

[16] Sloboda N, Sorlin A, Valduga M, Beri-Dexheimer M, Bilbault C, Fouyssac F et al. Deletion of chr7p22 and chr15q11: two familial cases of immune deficiency: extending the phenotype toward dysimmunity. Front Immunol [Internet]. 2019 [cited 2024 Mar 19];10:469055. Available from: www.frontiersin.org. 10.3389/fimmu.2019.01871 PMC6707040 31474980

[17] Lee SH, Byeon JH, Kim GH, Eun BL, Eun SH. Epilepsy in children with a history of febrile seizures. Korean J Pediatr [Internet]. 2016 [cited 2024 Mar 19];59:74. Available from: https://www.ncbi.nlm.nih.gov/pmc/articles/PMC4781735/10.3345/kjp.2016.59.2.74 PMC4781735 26958066

[18] Bando SY, Bertonha FB, Menezes PHN, Takahara AK, Khaled NA, Santos P et al. Transcriptomic analysis reveals distinct adaptive molecular mechanism in the hippocampal CA3 from rats susceptible or not-susceptible to hyperthermia-induced seizures. Scientific Reports 2023 13:1 [Internet]. 2023 [cited 2024 Mar 19];13:1–18. https://www.nature.com/articles/s41598-023-37535-w 10.1038/s41598-023-37535-w PMC10290664 37355705

[19] Kumar P, Lim A, Hazirah SN, Chua CJH, Ngoh A, Poh SL et al. Single-cell transcriptomics and surface epitope detection in human brain epileptic lesions identifies pro-inflammatory signaling. Nature Neuroscience 2022 25:7 [Internet]. 2022 [cited 2024 Mar 19];25:956–66. https://www.nature.com/articles/s41593-022-01095-5 10.1038/s41593-022-01095-5 PMC9276529 35739273

[20] Rana A, Musto AE. The role of inflammation in the development of epilepsy. Journal of Neuroinflammation 2018 15:1 [Internet]. 2018 [cited 2024 Mar 19];15:1–12. https://jneuroinflammation.biomedcentral.com/articles/10.1186/s12974-018-1192-7 10.1186/s12974-018-1192-7 PMC5952578 29764485

[21] Jiang NM, Cowan M, Moonah SN, Petri WA. The impact of systemic inflammation on neurodevelopment. Trends Mol Med [Internet]. 2018 [cited 2024 Mar 19];24:794. Available from: https://www.ncbi.nlm.nih.gov/pmc/articles/PMC6110951/10.1016/j.molmed.2018.06.008 PMC6110951 30006148

[22] Bach AM, Xie W, Piazzoli L, Jensen SKG, Afreen S, Haque R et al. Systemic inflammation during the first year of life is associated with brain functional connectivity and future cognitive outcomes. Dev Cogn Neurosci [Internet]. 2022 [cited 2024 Mar 19];53:101041. Available from: https://pubmed.ncbi.nlm.nih.gov/34973509/10.1016/j.dcn.2021.101041 PMC8728426 34973509

[23] Stebbing MJ, Cottee JM, Rana I. The role of ion channels in microglial activation and proliferation - a complex interplay between ligand-gated ion channels, K + channels, and intracellular Ca2+. Front Immunol [Internet]. 2015 [cited 2024 Mar 19];6:155959. https://www.frontiersin.org/journals/immunology/articles/10.3389/fimmu.2015.00497/full 10.3389/fimmu.2015.00497 PMC4617059 26557116

[24] Mango D, Nisticò R. Neurodegenerative Disease: What Potential Therapeutic Role of Acid-Sensing Ion Channels? Front Cell Neurosci. 2021;15:730641. https://www.ncbi.nlm.nih.gov/pmc/articles/PMC8531221/10.3389/fncel.2021.730641 PMC8531221 34690702

[25] Hanley SE, Cooper KF. Sorting Nexins in Protein Homeostasis. Cells [Internet]. 2021 [cited 2024 Mar 19];10:1–26. https://www.ncbi.nlm.nih.gov/pmc/articles/PMC7823608/10.3390/cells10010017 PMC7823608 33374212

[26] Yang J, Villar VAM, Rozyyev S, Jose PA, Zeng C. The emerging role of sorting nexins in cardiovascular diseases. Clin Sci (Lond) [Internet]. 2019 [cited 2024 Mar 19];133:723. https://pubmed.ncbi.nlm.nih.gov/30877150/10.1042/CS20190034 PMC6418407 30877150

[27] Tessier A, Callier P, LeMeur N, Frebourg T, Sabourin JC, Patrier S. Postmortem Diagnosis of Heart-hand Syndrome Associated With a 7p22.1p22.3 Deletion in a 16-week-old Fetus. Pediatr Dev Pathol [Internet]. 2019 [cited 2024 Mar 19];22:146–51. https://pubmed.ncbi.nlm.nih.gov/30193563/10.1177/1093526618799293 30193563

[28] Wolf DA, Lin Y, Duan H, Cheng Y. eIF-Three to Tango: emerging functions of translation initiation factor eIF3 in protein synthesis and disease. J Mol Cell Biol [Internet]. 2020 [cited 2024 Mar 19];12:403–9. 10.1093/jmcb/mjaa018 10.1093/jmcb/mjaa018 PMC7333474 32279082

[29] Sadato D, Ono T, Gotoh-Saito S, Kajiwara N, Nomura N, Ukaji M et al. Eukaryotic translation initiation factor 3 (eIF3) subunit e is essential for embryonic development and cell proliferation. FEBS Open Bio [Internet]. 2018 [cited 2024 Mar 19];8:1188–201. Available from: https://onlinelibrary.wiley.com/doi/full/10.1002/2211-5463.12482 10.1002/2211-5463.12482 PMC6070656 30087825

[30] Zhuo W, Chen J, Jiang S, Zheng J, Huang H, Xie P et al. Proteomic profiling of eIF3a conditional knockout mice. Front Mol Biosci [Internet]. 2023 [cited 2024 Mar 19];10. https://pubmed.ncbi.nlm.nih.gov/37152897/10.3389/fmolb.2023.1160063 PMC10154561 37152897

[31] Sahara M, Santoro F, Sohlmér J, Zhou C, Witman N, Leung CY et al. Population and Single-Cell Analysis of Human Cardiogenesis Reveals Unique LGR5 Ventricular Progenitors in Embryonic Outflow Tract. Dev Cell [Internet]. 2019 [cited 2024 Mar 19];48:475–490.e7. https://pubmed.ncbi.nlm.nih.gov/30713072/10.1016/j.devcel.2019.01.005 30713072

[32] Rüdebusch J, Benkner A, Poesch A, Dörr M, Völker U, Grube K et al. Dynamic adaptation of myocardial proteome during heart failure development. PLoS One [Internet]. 2017 [cited 2024 Mar 19];12:e0185915. https://journals.plos.org/plosone/article?id=10.1371/journal.pone.0185915 10.1371/journal.pone.0185915 PMC5626523 28973020

[33] Xia Z, Tong X, Liang F, Zhang Y, Kuok C, Zhang Y et al. Eif3ba regulates cranial neural crest development by modulating p53 in zebrafish. Dev Biol [Internet]. 2013 [cited 2024 Mar 19];381:83–96. https://pubmed.ncbi.nlm.nih.gov/23791820/10.1016/j.ydbio.2013.06.009 23791820

[34] Erhardt S, Zheng M, Zhao X, Le TP, Findley TO, Wang J. The Cardiac Neural Crest Cells in Heart Development and Congenital Heart Defects. Journal of Cardiovascular Development and Disease. 2021, Vol 8, Page 89 [Internet]. 2021 [cited 2024 Mar 19];8:89. https://www.mdpi.com/2308-3425/8/8/89/htm 10.3390/jcdd8080089 PMC8397082 34436231

[35] Plein A, Fantin A, Ruhrberg C. Neural crest cells in cardiovascular development. Curr Top Dev Biol [Internet]. 2015 [cited 2024 Mar 19];111:183–200. https://pubmed.ncbi.nlm.nih.gov/25662261/10.1016/bs.ctdb.2014.11.006 25662261

[36] Riolo G, Cantara S, Ricci C. What’s wrong in a jump? Prediction and validation of splice site variants. Methods Protoc. 2021;4. 10.3390/mps4030062. 10.3390/mps4030062 PMC8482176 34564308

[37] Zeng Z, Bromberg Y. Predicting Functional effects of Synonymous variants: a systematic review and perspectives. Front Genet. 2019;10. 10.3389/fgene.2019.00914. 10.3389/fgene.2019.00914 PMC6791167 31649718

[38] Bromberg Y, Kahn PC, Rost B. Neutral and weakly nonneutral sequence variants may define individuality. Proc Natl Acad Sci U S A. 2013;110. 10.1073/pnas.1216613110. 10.1073/pnas.1216613110 PMC3761624 23940345

[39] Xiong HY, Alipanahi B, Lee LJ, Bretschneider H, Merico D, Yuen RKC, et al. The human splicing code reveals new insights into the genetic determinants of disease. Science. 1979;2015347. 10.1126/science.1254806. 10.1126/science.1254806 PMC4362528 25525159

